# Effectiveness of a nurse-led, community-based frailty prevention program for prefrail older adults: a pragmatic quasi-experimental trial

**DOI:** 10.1186/s12912-025-04131-4

**Published:** 2025-12-12

**Authors:** Dong-Ok Lee, Jina Choo, Songwhi Noh, Yura Shin

**Affiliations:** 1 College of Nursing, Korea University, Seoul, 02841 South Korea; 2 Transdisciplinary Major in Learning Health Systems, Graduate School, Korea University, Seoul, 02841 South Korea

**Keywords:** Frail elderly, Community health services, Public health nursing, Community health workers, Program evaluation

## Abstract

**Background:**

Prefrail older adults face a higher risk of progressing to frailty, and preventing this progression may reduce mortality. However, evidence on the effectiveness of nurse-led, community-based programs with an integrated approach remains limited. We aimed to develop and evaluate the effectiveness of a nurse-led frailty prevention (NurFP) program in prefrail older adults living in the community. The program was developed based on an ecological framework that addresses frailty risk factors and corresponding intervention strategies across intrapersonal, interpersonal, and community levels, and incorporates a nurse-led multidisciplinary approach.

**Methods:**

A mixed-methods, quasi-experimental, pragmatic trial was conducted using a pretest-posttest design over a 12-week interval. Participants were forty prefrail older adults residing in Seoul, South Korea. Four administrative units within J district in Seoul were pair-matched based on community characteristics, with two units assigned to the NurFP group and the remaining two to the control group. Participants were recruited from each of NurFP (*n* = 20) and control (*n* = 20) groups using convenience sampling. The NurFP group received a 12-week program with main intervention modes of group education and walking sessions; the control group received a single health counselling session. Primary outcome was frailty score; secondary outcomes were intrapersonal, interpersonal, and community-level risk factors for frailty. Outcomes were measured via self-reported questionnaires, of which the community-level factor was assessed to explore perceived supportive environment for frailty prevention using focus group interview following the intervention.

**Results:**

Compared to the control group, the NurFP group demonstrated a significant reduction in frailty scores (*p* = .025), with notable improvements in the physical (*p* = .037) and social (*p* = .020) domains. The NurFP group exhibited greater improvements in intrapersonal-level factors, including adherence to physical activity (*p* < .001) and chronic disease self-management (*p* < .001), as well as in interpersonal-level factors such as perceived social support (*p* < .001). Focus group interviews revealed heightened awareness of supportive community environments for frailty prevention among the NurFP group and their key stakeholders.

**Conclusions:**

The NurFP program may improve frailty and its ecological multi-level risk factors and serve as a feasible community-based intervention model for frailty prevention.

**Trial registration:**

This study has been retrospectively registered with the Clinical Research information Service (CRIS) of South Korea on May 24, 2025 (KCT0010511).

**Supplementary Information:**

The online version contains supplementary material available at 10.1186/s12912-025-04131-4.

## Background

Frailty is a multidimensional condition characterized by physical, psychological, and social decline, reflecting a reduced capacity to independently perform daily activities [1]. Globally, approximately 11–12% of older adults are classified as frail [2]. Frailty is strongly associated with increased risks of hospitalization and mortality among older adults [3–7]. Evidence suggests that frail older individuals are 1.56 times more likely to be hospitalized [8], and have a sixfold higher mortality risk within three years compared to their non-frail counterparts [9].

Prefrailty refers to an intermediate stage preceding the onset of frailty [1]. Approximately 39–42% of older adults are considered prefrail [2]. Prefrail individuals may have more than twice the risk of progressing to frailty within three years compared to their non-prefrail counterparts [3]. Preventing this progression may reduce mortality by 3–5% among older adults [10]. In particular, rapidly aging societies such as South Korea face an increasing burden of frailty. In this context, the development of an effective healthcare system emphasizing early detection and prevention of prefrailty is an urgent priority.

Since 2009, the Ministry of Health and Welfare in South Korea has officially launched a model of the Visiting Health Management Program [11], through which public health centers in each autonomous district began offering free home-visiting nursing services to vulnerable populations. In 2015, the Seoul Metropolitan Government expanded upon this model by introducing a more community-centered approach, assigning community health nurses to local administrative community service centers to enhance accessibility and universal access [12, 13]. Under this new model, nurses have been encouraged not only to provide conventional outreach and home-visiting services, but also to proactively initiate and promote small-scale, group-based programs conducted at community service centers—such as frailty prevention interventions—as part of their expanded roles [14].

A recent study in the UK demonstrated that nurses played a leading role in assessing patients’ frailty by collaborating with patients, families, and other healthcare professionals throughout various stages of the care process [15]. Salem et al. further emphasized that nurses, as front-line experts caring for vulnerable population groups, should actively raise awareness and work to identify and prevent frailty risk factors among older adults in the community [16]. In this context, it is crucial for community health nurses to detect and manage frailty-related risks at the earliest possible stage [17].

Community health nurses should employ an integrated approach to comprehensively identify risk factors for frailty and effectively prevent it among community-dwelling older adults. For this approach, Salem et al. [16] addressed a systematic, theoretical basis by adopting Gobbens et al.’s multidimensional concept of frailty [1] and proposed a comprehensive framework of risk factors for frailty, including individual-level factors (e.g., age, gender), situational factors (e.g., life events), health-related factors (e.g., chronic diseases, malnutrition), health behavior factors (e.g., smoking), resource factors (e.g., social support), physiological factors (e.g., inflammation), and environmental factors (e.g., physical living conditions) [16, 18]. However, most studies have focused primarily on physical frailty, with limited evidence regarding the effectiveness of nurse-led, preventive and comprehensive interventions targeting multiple risk factors for frailty [19].

**Fig. 1:**
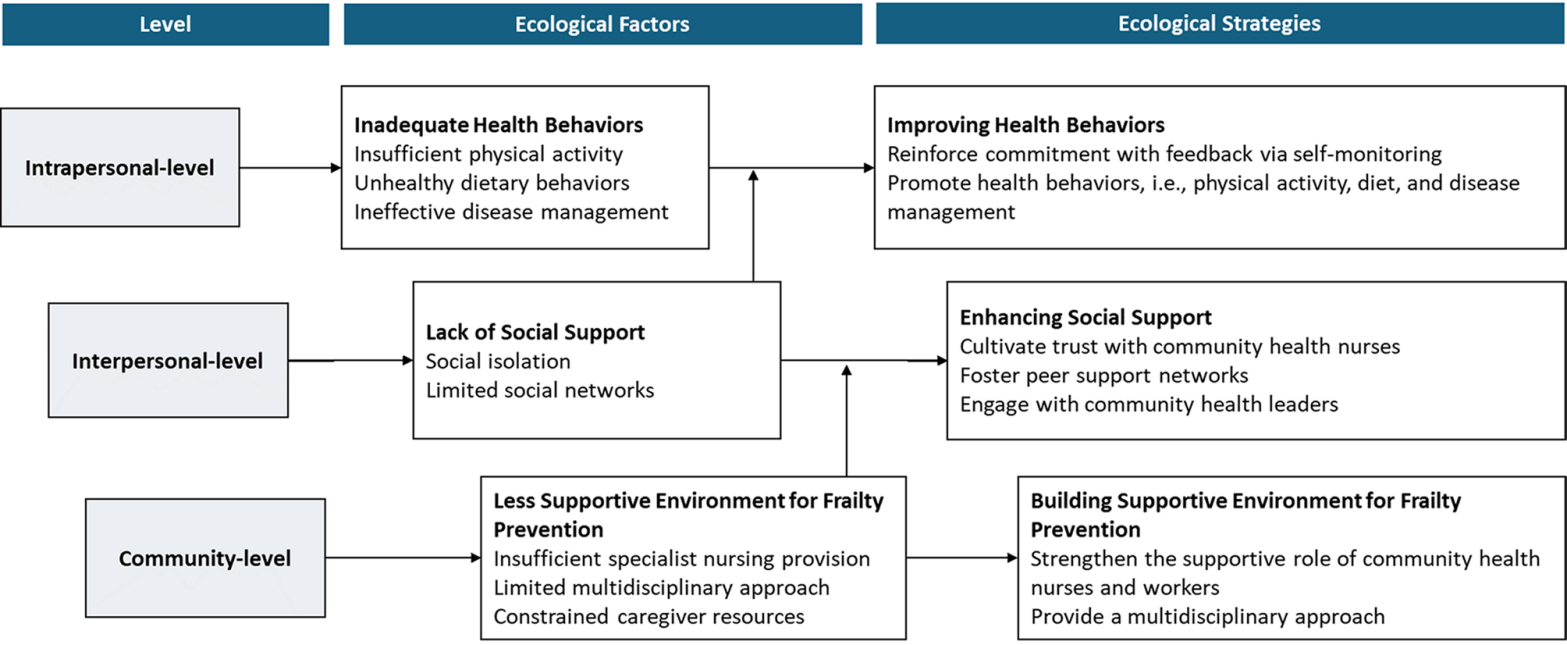
Ecological risk factors and intervention strategies for pre-frail older adults living in the community

To build upon the comprehensive framework of frailty risk factors proposed by Salem et al. [16], the present study integrates McLeroy et al.’s ecological perspective [20], which offers a theoretical foundation for developing multi-level strategies to prevent and mitigate health problems at the population level. This perspective also emphasizes the identification of risk factors across multiple levels—intrapersonal, interpersonal, and community—and the design of corresponding interventions tailored to each level. Grounding intervention strategies in McLeroy et al.’s ecological model (1988) may offer several key advantages in designing effective and sustainable frailty prevention programs [20]. First, by addressing multiple levels of influence, this ecological approach increases the likelihood of achieving sustainable frailty-related behavior change, particularly beyond the scope of individual-level interventions. Second, the model facilitates the integration of diverse stakeholders—including healthcare professionals, community health workers, and policymakers—thereby enhancing program feasibility, acceptability, and long-term scalability. Third, ecological strategies are well-suited to advancing health equity, as they account for structural and environmental determinants that disproportionately affect populations vulnerable to frailty. Thus, the ecological model provides a robust theoretical foundation for frailty prevention interventions aiming to address frailty-related risk factors and behaviors among vulnerable individuals within a broader social and environmental context. In alignment with this ecological model, health behavior factors (e.g., physical activity, nutrition) may be situated at the intrapersonal-level, resource factors (e.g., social support) at the interpersonal-level, and environmental factors (e.g., physical and social environments) at the community-level. Based on this, we have identified intrapersonal, interpersonal, and community-level risk factors associated with frailty (Fig. 1) and developed corresponding intervention strategies for each level in the present study. Inadequate health behaviors—such as insufficient physical activity [21], unhealthy dietary behaviors [22], and ineffective chronic disease self-management [23]—were classified as intrapersonal-level risk factors. Resource-related factors, including lack of social support [24], were categorized as interpersonal-level risk factors. At the community-level, environmental factors such as less supportive environment for frailty prevention [25, 26] were conceptually reframed as community-level risk factors.

Based on these multi-level classifications, we developed tailored solution strategies: improving health behaviors at the intrapersonal level, enhancing social support at the interpersonal level, and building a supportive environment for frailty prevention at the community level. These strategies were implemented through a multidisciplinary approach [27] involving community health nurses, local experts, and community health workers, under the coordination of a nurse-led care model. Using group dynamics through community participation, group education and activities along with individualized counselling may effectively improve frailty among older adults in community settings [28, 29]. In this context, the present study designed a nurse-led, community-based frailty prevention (NurFP) program with a multidisciplinary approach through group education and activities. Finally, this study aims to evaluate the effectiveness of the NurFP program in improving frailty and its associated ecological multi-level risk factors among community-dwelling older adults.

## Methods

### Study design

**Fig. 2:**
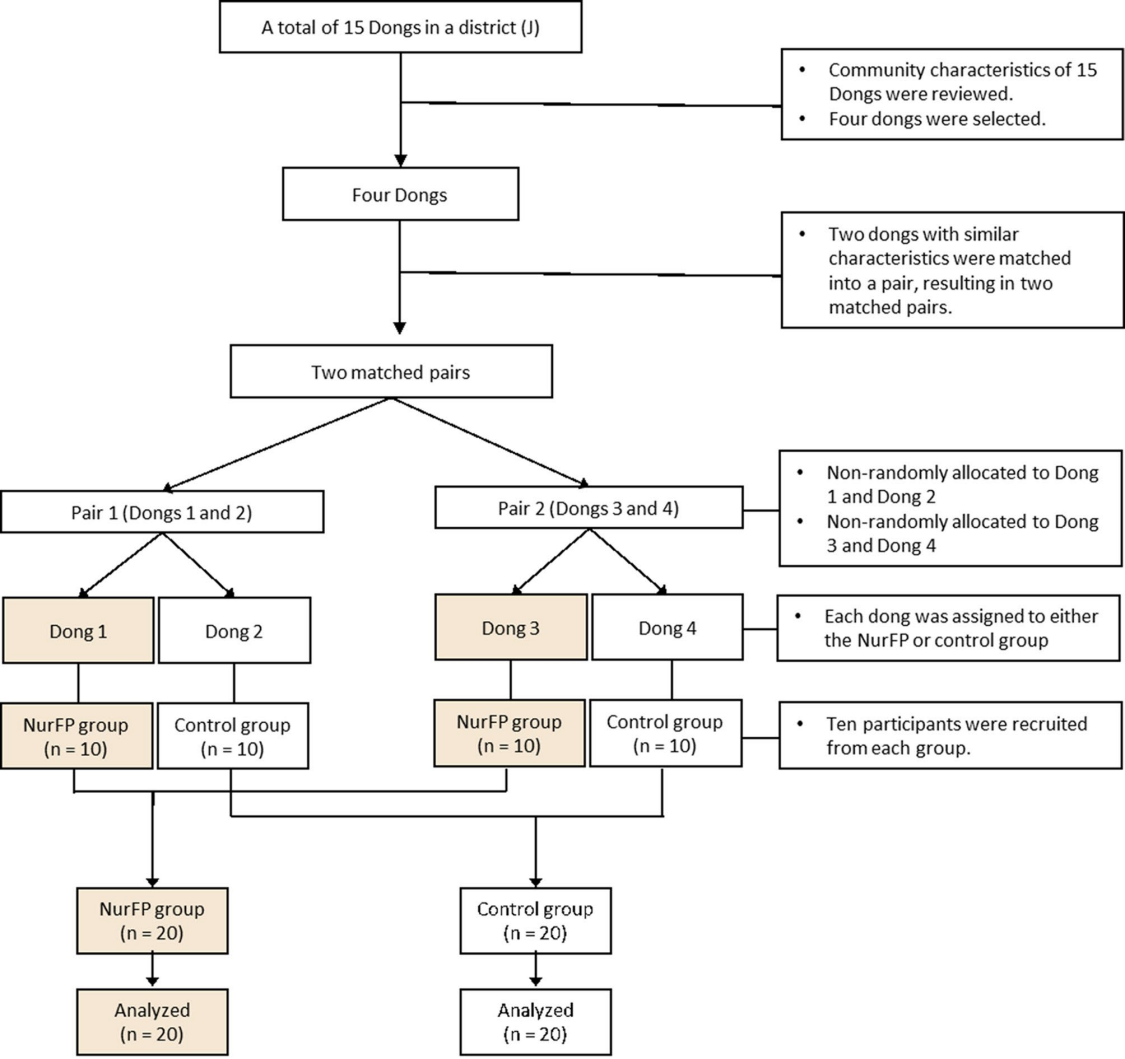
Participant flow in the study. NurFP = nurse-led frailty prevention. Note. A ‘Dong’ is an administrative unit within a district in South Korea, similar to a neighborhood or precinct, used for local governance and public services

This study employed a quasi-experimental, pragmatic clinical trial design based on a mixed-methods approach to evaluate the effectiveness of the 12-week NurFP program. Four administrative units (i.e., dongs), called as dongs in South Korea, were selected within J autonomous district in Seoul and pair-matched based on community characteristics, with two dongs allocated to the intervention group (i.e., NurFP group) and the remaining two to the control group (Fig. 2). The NurFP group participated in the 12-week NurFP program, while the control group received a single health counselling session. Pre- and post-tests for measurements were conducted at a 12-week interval. Quantitative data were collected through self-administered questionnaires at both pre- and post-tests. Qualitative data were collected through focus group interviews (FGIs) conducted after the completion of the intervention with three groups: participants in the NurFP program (*n* = 5), community health nurses (*n* = 5), and community health workers (*n* = 3). The inclusion of qualitative methods aimed to capture in-depth perspectives on the supportive environment for frailty prevention—an important community-level factor that may not be sufficiently explored through quantitative methods alone—and to further explore stakeholders’ perceptions of the overall NurFP program as an available personnel resource for providing community support to clients, within the framework of the ecological model.

As this study adopted a pragmatic trial design in community settings, double blinding was not feasible due to the nature of the interventions and community practice environments. In this context, neither study participants nor assessors were blinded to the study groups. Nevertheless, several measures were implemented to minimize potential observer bias and placebo effects. Specifically, anthropometric measurements were conducted by trained personnel from an accredited institution using standardized and validated instruments [30]. Questionnaires were administered by five community health nurses and researchers who were briefly instructed prior to data collection. In addition, participants were not informed of the specific study hypotheses.

### Study participants

A total of 40 older adults participated in the present study (Fig. 2), with 20 assigned to the NurFP group and 20 to the control group through non-random allocation. The inclusion criteria for study participants were as follows: (1) aged 65 years or older, (2) residing in J district, one of 25 autonomous districts in Seoul, (3) classified as pre-frail, (4) having no severe cognitive impairment (defined as a Mini-Mental State Examination for Dementia Screening [MMSE-DS] score of 18 or higher), and (5) able to communicate verbally and cooperate with study assessments. The prefrail condition was classified based on scores obtained from the Korean version of the Kihon Checklist [31], which has been validated [32] and subsequently adopted for use in public health centers across South Korea. The Checklist comprises 28 items, with a total score ranging from 0 to 31. In this study, a score between 4 and 12 was used to define the prefrail condition. The exclusion criteria included individuals who were already participating in other health promotion programs at the time of recruitment or those who planned to engage in similar programs during the intervention period.

The **sample size** for this study was calculated using the G^*^Power 3.1 program. Based on a previous study [33], the effect size was set at Cohen’s *d* = 1.32, with a significance level (α) of 0.05 and a power (1 − β) of 0.95. The minimum sample size was calculated as 16 participants per group, totaling 32 participants. Considering an anticipated dropout rate of 20%, the target sample size was adjusted to 40, with 20 participants allocated to each of the intervention and control groups.

#### Recruitment of study participants and group allocation

We obtained approval to conduct the study from the director of the public health center in J district, Seoul. After then, we reviewed six community characteristics—population size, prevalence of diagnosed diseases, self-rated health, neighborhood environment, health behaviors, and housing types—based on data from the 2014–2017 Korea Community Health Survey [34] and the National Statistical Office [35]. Based on the review and expert consultation, four administrative dongs out of 15 in J district were selected. A ‘dong’ is an administrative unit within a district in South Korea, similar to a neighborhood or precinct, used for local governance and public services. Four dongs were matched into two pairs based on the six previously described community characteristics to ensure balanced similarity between the pairs. Each dong in a pair was evaluated, through expert consultation, as being at a “good,” “moderate,” or “low” level for each of the six community characteristics (Supplementary Table 1).

Next, to achieve a balanced distribution of community characteristics, one dong from each matched pair was selected and allocated to either the NurFP or control group using a cluster allocation strategy. Random assignment within each pair was not feasible. In Pair 1, one dong (“Dong 1”) had already met the physical infrastructure requirements (e.g., availability of classrooms and exercise rooms) necessary to implement the NurFP program. As a result, Dong 1 was allocated to the NurFP group, and the other dong (“Dong 2”) was assigned to the control group. In Pair 2, the dong selected for the NurFP group (“Dong 3”) was chosen based on its geographical proximity to Dong 1, to enhance accessibility and convenience for participants. Ultimately, ten participants (*n* = 10) were recruited from each of the four administrative dongs, resulting in a total sample of 40 participants (Fig. 2). Recruitment was conducted by community health nurses assigned to each administrative dong, who invited residents under their care to participate and enrolled them on a first-come, first-served basis. Following approval from the local community (dong) service centers in each dong, participants were recruited via bulletin board notices and informational flyers. The recruitment of the participants was conducted from October 1, 2019 to October 3, 2019.

The FGI participants of 13 individuals were recruited to explore perceptions of changes in perceived supportive environment for frailty prevention. Of the 13 participants, five in the NurFP group (*n* = 5), three community health workers (*n* = 3), and five community health nurses (*n* = 5) were recruited. The inclusion criteria for each group were as follows: NurFP group participants (*n* = 5) were purposively selected from the NurFP group who completed the full 12-week intervention and expressed willingness to participate in the FGI. Community health workers (*n* = 3) were lay residents of J district who had obtained walking leader certification and voluntarily facilitated group walking sessions for 12 weeks as part of the program. Community health nurses (*n* = 5) were those who were assigned to the two administrative dongs in the NurFP group and also served as program instructors.

### Development and implementation of the NurFP program

#### Core principles of the NurFP program

The NurFP program was designed to prevent frailty among community-dwelling older adults, grounded in Salem et al.’s framework of multiple frailty risk factors and McLeroy et al.’s ecological model of multilevel factors and corresponding intervention strategies (Fig. 1). A multidisciplinary approach was employed throughout the program design and implementation. To address the multi-level risk factors associated with frailty, the following **intervention strategies** were formulated: (1) improving health behaviors at the intrapersonal level (2) enhancing social support at the interpersonal level, and (3) building a supportive environment for frailty prevention at the community-level.

**Fig. 3:**
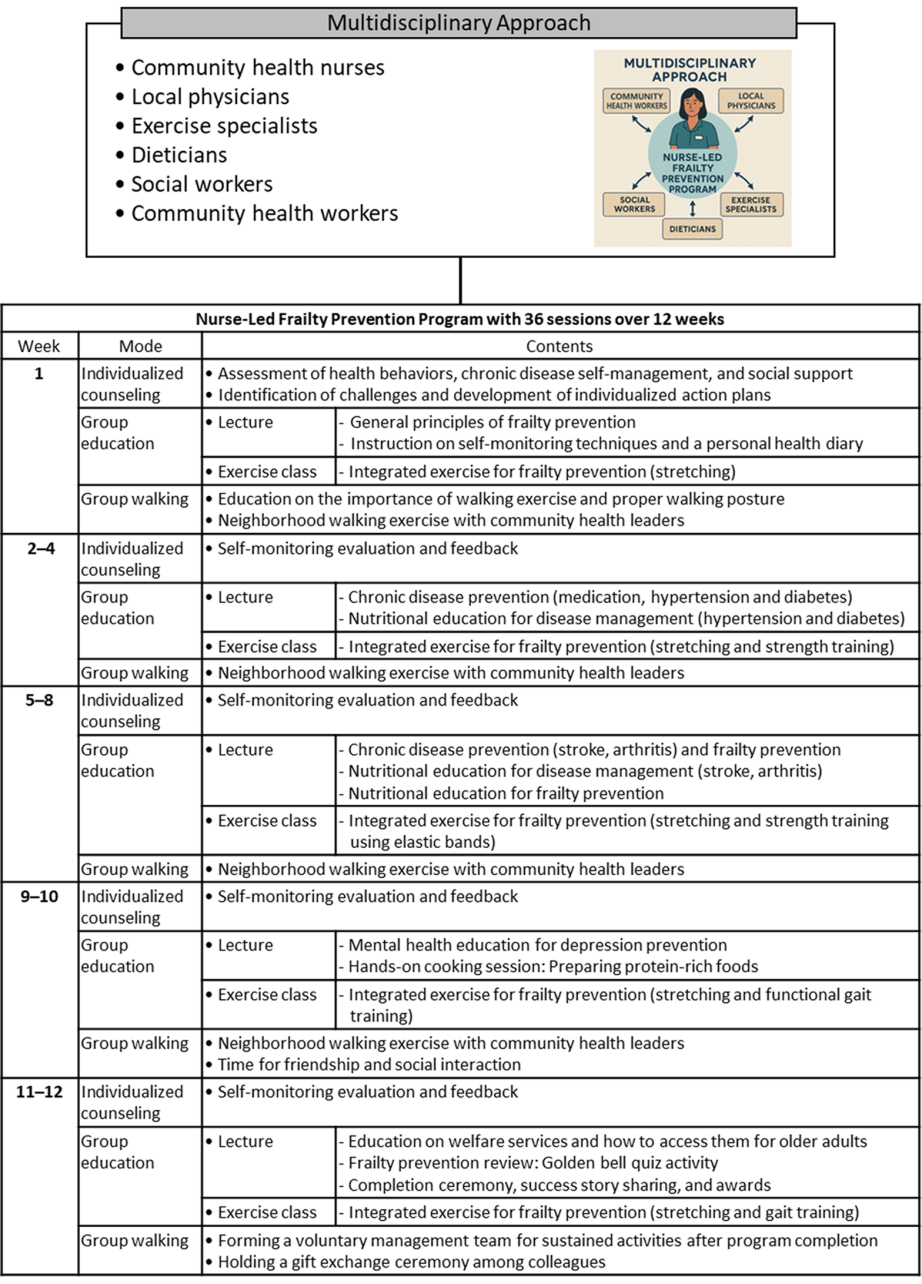
Structure of the 12-week of Nurse-led Frailty Prevention program

The strategies for health behaviors consisted of reinforcing commitment with feedback via self-monitoring on behavioral changes and enhancing physical activity, healthy dietary behaviors, and disease management. Participants were guided to recognize that healthy dietary behaviors and physical activity are grounded in self-care and were encouraged to practice these behaviors daily at home, rather than limiting them to the program sessions. During their weekly visits to the community service center for the NurFP program sessions, the assigned nurse reviewed the participants’ self-monitoring records with them prior to each session. The strategies for enhancing social support comprised cultivating trust with nurses, fostering peer support networks, and engaging with community health workers. The strategies for building supportive environment for frailty prevention included increasing the awareness of NurFP participation and strengthening the supportive role of community health nurses and workers. **A multidisciplinary approach** was employed to deliver group education and walking sessions in the NurFP program (Fig. 3), involving a team composed of one researcher (the first author), five community health nurses, two professors of community health nursing, one local physician, one dietitian, one exercise specialist, one social worker, and three community health workers. Each team member had a minimum of five years of professional experience in their respective field.

#### Fidelity of the NurFP program

The development of strategies for the NurFP program was grounded in a comprehensive literature review and a systematic needs assessment. The intervention was further refined through content validity with expert consultation and a pilot test. Content validity was evaluated by a panel of six experts, comprising two professors of community health nursing, one professor specializing in physical activity, one nutrition expert, and two practicing community health nurses. The average content validity index across intervention components was 0.86, indicating an acceptable level of validity.

Next, a pilot test was conducted with six pre-frail older adults who comprised a separate group excluded from both the NurFP and control groups. This pilot test aimed to assess the program’s clarity, acceptability, and level of participant engagement. To ensure consistency in program delivery, two types of **educational materials** were developed: an ‘Educational Manual for Nurse-led Frailty Prevention’ intended for nurses, and a ‘Health Diary for Self-Monitoring’ designed for participants in the NurFP group. These materials were rigorously consolidated following a thorough review by two professors of community health nursing and one PhD-level consultant. Moreover, to ensure that both the overall program and the content of each session were implemented according to the planned protocol, the first author participated in every session and closely monitored the implementation process.

Prior to the implementation of the 12-week NurFP program, **professional training** was provided to enhance nurses’ competencies in delivering frailty prevention interventions. Five community health nurses—assigned to the two administrative dongs selected for the NurFP group—participated in a 24-hour training program delivered over three consecutive days (eight hours per day). These nurses served as the core implementers of the nurse-led intervention, which included individualized counseling, coordination of group education, and facilitation of group walking sessions (Fig. 3). The training curriculum (Supplementary Table 2) was developed through a comprehensive literature review and a needs assessment focused on frailty risk factors and prevention strategies. Anchored in the ecological model, the educational content was organized into an introductory session, followed by sessions at the intrapersonal, interpersonal, and community levels. At each level, the sessions addressed the multidimensional nature of frailty—encompassing physical, psychological, and social domains—and the role of visiting nurses in comprehensive prevention. The content also integrated practical skills and strategies, including frailty assessment, intervention, community participation and collaboration, and multidisciplinary approach, for frailty prevention and management. An expert panel—including professors specializing in community health nursing—collaborated with the research team to design and validate the curriculum. Instructional methods included lectures, demonstrations, hands-on practice, and group discussions to promote practical skill development and active engagement. Formal inter-rater reliability testing of the intervention, which was implemented by community health nurses and health workers, could not be conducted due to the real-world nature of the community-based setting. However, throughout the 12-week intervention, the first author provided supervision and maintained continuous communication with the five community health nurses and community health workers involved to ensure adherence to the protocol and to promptly address any deviations.

Additionally, three community health workers were involved in the program to support physical activity sessions. These leaders were affiliated with a public health center in J district, where the study participants were recruited. They had been selected through a local health promotion initiative focused on physical activity and had completed a 10-week training course [36]. All three held official walking leader certifications from the Korea Walking Association [37].

#### Implementation of the NurFP program

The NurFP program consisted of 36 sessions delivered over 12 weeks (Fig. 3), with three structured sessions held each week: individualized counseling, group education (lecture and exercise class), and group walking.

A weekly 10-minute individualized counseling session was provided by five nurses every Monday in an education room at a community service center in J district. During each session, nurses reviewed participants’ 7-day self-monitoring records on physical activity and healthy dietary behaviors, facilitated recall for any missed entries, and provided tailored feedback.

Following the session, a 100-minute group education session was conducted, comprising a 50-minute lecture and a 50-minute exercise class. Each lecture began with senior icebreaking activities, such as group singing, a name game, partner massage, and a clapping game which were facilitated by the first author. Following these activities, the lecture corresponding to the week’s topic was delivered by a relevant expert. The lectures were coordinated by two of the five community health nurses at the administrative dong and delivered by the multidisciplinary team, while the exercise classes were led by an exercise specialist. The lecture for healthy dietary behaviors was delivered primarily by community health nurses, with support from a registered dietitian, using food models, video materials, and adapted songs with familiar melodies to enhance comprehension among older adults. Lectures for chronic disease self-management were addressed an integrated range of topics, including symptom recognition and management, treatment and medication adherence, lifestyle modification, and linkage to family and community resources.

The exercise class for physical activity enhancement was implemented by an exercise specialist through a warm-up (10 min), guided stretching (10 min), muscle-strengthening exercises (10 min), functional gait training (10 min), and a cool-down (10 min), supplemented by printed materials. The muscle-strengthening exercises incorporated bodyweight-based resistance training and partner-assisted functional movements using elastic resistance bands. These consisted of six structured modes targeting both upper and lower body strength, as well as balance and coordination. These included punch greeting, rowing together, open-arm hug, sit-to-stand with partner support, heel raises, and single-leg balance. Exercise intensity was maintained at a low to moderate level, appropriate for pre-frail older adults, and was individually adjusted based on each participant’s physical capacity and health status. Each exercise lasted 1 min and 40 s and consisted of 20 repetitions.

In addition, a 60-minute neighborhood walking session, led by three community health workers, was held every Thursday as part of the group-based intervention. The activity took place on a designated walking trail near J district that was free of vehicular traffic and easily accessible to participants. Among the facilitators, the most experienced community health worker served as the lead guide, while the other two were positioned at the midpoint and rear of the walking formation to provide supervision and ensure safety. Participants were instructed to bring appropriate walking gear, including athletic shoes, cushioned socks, gloves, hats, and water bottles. For ease of interpersonal identification, each participant was required to wear a name tag during every session. Each walking session followed a standardized protocol: participants first engaged in greetings and 10 min of warm-up stretching exercises, followed by 40 min of continuous walking, and concluded with 10 min of cool-down stretches.

### Data collection

Quantitative and qualitative data were collected at baseline and after the implementation of the NurFP program by an assessment team comprising a researcher (i.e., the first author) and five nurses. Data collection for the follow-up assessment was conducted from January 7 to 9, 2020. Prior to both the pre- and post-tests, standardized training sessions were provided to ensure consistency in data collection procedures across all team members. For the quantitative evaluation, a pre-test was conducted over three days prior to the start of the program, and a post-test was administered over three days following the completion of the 12-week intervention.

To assess participants’ perceptions of the community-level “supportive environment for frailty prevention”, FGIs were held with three sessions of each stakeholder group after the intervention. The focus group interviews were organized into four components: introductory questions, transition questions, key questions, and concluding questions. For nurses and community health workers, the key questions explored their perceptions of the NurFP program’s impact on older adults’ health and its specific effects on frailty prevention. Additional questions addressed the perceived need for program expansion, the benefits of walking groups for frailty prevention, and their confidence in independently leading the program (e.g., “Do you think you have gained the confidence to take a leading role in operating the NurFP program?”). For older adults in the NurFP group, the questions focused on subjective experiences with the program, including perceived health benefits, social connectedness, mutual exchange of health-related information, and reduced feelings of loneliness. Participants were also asked about their willingness to rejoin the program and recommend it to others (e.g., “To what extent do you think participating in the NurFP program has benefited your health?”).

Each session lasted approximately 60 to 70 min and was conducted in a designated meeting room within the local community service center to ensure a comfortable and familiar environment. The first author facilitated the interviews as the moderator, while an assistant researcher managed audio recording and field notes. All interviews were audio-recorded with participants’ informed consent and transcribed within three days.

### Measures

Participants’ general characteristics as well as measures of primary and secondary outcomes were measured. The primary outcome was the score of frailty, while the secondary outcomes comprised multi-level risk factors for frailty.

#### Participants’ general characteristics

General characteristics of participants were assessed as follows. Sociodemographic characteristics comprised age, gender, marital status, education years, perceived economic status, and whether the individual lived alone [38]. Health-related characteristics included self-rated health, number of chronic diseases, oral health problems, current smoking, alcohol drinking, exercise experience, body mass index (kg/m^2^), and cognitive function. Self-rated health was assessed as poor vs. good. Exercise experience was assessed based on whether participants had ever engaged in exercise for more than 30 min on a regular basis. Height and weight were measured using a stadiometer and a digital scale, respectively. Cognitive function was assessed only at pre-test, using the MMSE-DS to determine participants’ eligibility for inclusion in the study [39].

#### Primary outcome: frailty

Frailty was measured using the Tilburg Frailty Indicator (TFI), a validated instrument developed by Gobbens et al. in 2010 [18]. The TFI is considered as an appropriate tool for identifying frailty in community-dwelling older adults, as it comprehensively addresses physical, psychological, and social domains. The TFI consists of Part A and Part B. In this study, we used Part B, a self-reported frailty scale comprising 15 items across three domains: the physical domain (8 items; score range: 0–8), the psychological domain (4 items; 0–4), and the social domain (3 items; 0–3). The total score ranges from 0 to 15, with higher scores indicating greater frailty. The Cronbach’s alphas for the TFI in the previous study was 0.74 [40], while it was 0.85 in the present study.

#### Secondary outcomes: multilevel risk factors for frailty

##### Intrapersonal-level factors for frailty

Three key variables of adherence to physical activity, adherence to healthy dietary behaviors, and chronic disease self-management behaviors were included as the following. **Adherence to physical activity** was evaluated based on the fulfillment of walking recommendations. Specifically, the recommended threshold was set at 4,500 steps per day, based on the age-adjusted guideline for adults in their 70s as recommended by the American Heart Association [41]. Study participants were instructed to use the WalkOn mobile application to track their physical activity for seven consecutive days. The percentage of steps taken during the pre- and post-test periods was calculated relative to the recommended threshold using the following formula: (actual steps ÷ 4,500) × 100. Step count data were retrieved from the WalkON mobile application [42], which participants used to log their activity in a health diary. The averages over 7 days were computed, with higher scores indicating greater adherence to the recommended physical activity levels. **Adherence to healthy dietary behaviors** was measured using a self-assessment checklist. We used four items to assess daily protein intake, based on the question: “On average over the past three months, did you consume the following sources of protein each day?” Three items were adapted from the Mini Nutritional Assessment [43], which assess the consumption of (1) dairy products (e.g., milk, yogurt, cheese), (2) legumes or eggs, and (3) meat, fish, or poultry. An additional item was developed by the researchers to assess (4) the intake of protein supplements. Each item was answered using a binary response format (yes/no). Scoring was assigned as follows: 1 point for no intake at all; 2 points for intake on one item; 3 points for two items; 4 points for three items; and 5 points for all four items. The point was indicated as ‘dietary score’. The recommended threshold for the dietary score was set at an average of ≥ 3.5, which corresponds to 70% adherence—a level commonly regarded as the standard criterion for determining behavioral goal achievement in adherence studies [44, 45]. The percentage of adherence to the recommended dietary threshold was calculated using the formula: (actual dietary score ÷ 3.5) × 100. Daily dietary adherence scores were then computed as average values ranging from 0 to 100, with higher scores indicating greater adherence to healthy dietary behaviors. **Chronic disease self-management behaviors** were assessed using the Korean version of the Appraisal of Self-care Agency Scale-Revised, translated by Kim [46, 47]. The scale consists of 15 items, including four reverse-coded items, rated on a five-point scale (1 = strongly disagree to 5 = strongly agree). Total scores range from 15 to 75, with higher scores indicating stronger self-management. The Cronbach’s alpha in previous studies was reported as 0.79–0.90 [46, 47], while Cronbach’s alpha in the present study was 0.73.

##### Interpersonal-level factors for frailty

Perceived social support was measured using the Korean version of the Enhancing Recovery in Coronary Heart Disease Social Support Instrument [48], adapted by Jeon et al. [49] This instrument consists of six items encompassing emotional, informational, and instrumental support. The Korean version consists of six items, with one item removed from the original version, and demonstrated good content validity following a back-translation process [49]. Each item is answered with a binary response option: “Yes” (1 point) or “No” (0 points). The total score ranges from 0 to 6, with higher scores indicating greater levels of perceived social support. The Cronbach’s alpha in previous studies was reported as 0.86 [48], while Cronbach’s alpha in the present study was 0.81.

##### Community-level factors for frailty

Supportive environment for frailty prevention as community-level strategies was qualitatively assessed by using FGI. Supportive environments may mitigate social frailty by fostering social engagement, enabling early identification of pre-frail individuals, and supporting the effective delivery and diffusion of frailty prevention interventions [50, 51]. Given the inherent challenges in quantitatively capturing perceptions of community-level support, a qualitative approach was adopted to explore participants’ perceptions and experiences regarding the implementation and effectiveness of the NurFP program in real-world community settings. The FGIs included community health nurses, community health workers, and older adults who participated in the NurFP program, offering valuable insights into the feasibility, community engagement, and collaborative processes underpinning the intervention. Participants also reflected on the effectiveness of multidisciplinary group education, group walking activities, and the human resources of support—such as nurses, multidisciplinary experts, and peer support provided by community health workers. This qualitative inquiry elucidated contextual factors and practical challenges that could not be captured through quantitative measures, thereby enriching the overall interpretation of the program outcomes.

### Data analysis

The data were analyzed using SPSS version 29.0 [52]. Descriptive statistics—including frequencies, percentages, means, and standard deviations—were used to report participants’ general characteristics. To test baseline homogeneity between the NurFP and control groups, Chi-square test, Fisher’s exact test, independent-t test, and Mann–Whitney U test were performed according to the normality of variables. To examine difference in primary and secondary outcomes between pre- and post-test, the Wilcoxon signed-rank test was performed. To examine differences in mean differences (∆ = posttest – pretest), the Mann-Whitney U test was performed. Non-parametric tests, i.e., the Wilcoxon signed-rank test and the Mann–Whitney U test, were selected based on the rationale that the primary and secondary outcome variables did not meet the assumption of normality according to the Kolmogorov–Smirnov test.

Qualitative data from the FGIs were transcribed and analyzed using a content analysis approach, as described by Graneheim and Lundman [53]. To enhance the objectivity and neutrality of the analytic process, the first and second authors independently reviewed the interview transcripts and identified initial themes and categories. The emerging themes and categories were continuously discussed and refined through collaborative analysis until consensus between the authors was achieved.

### Ethical consideration

The study was conducted in accordance with the Helsinki Declaration of Ethical Principles for Medical Research, in which human subjects participate, and was approved by the Korea University Institutional Review Board (IRB) (No. KUIRB-2019-0247-01). Written informed consent was obtained from all participants prior to their participation in the study.

## Results

### Participants’ general characteristics and homogeneity between-groups

The mean age of participants (*N* = 40) was 77.7 years (Table 1). The majority were women (90.0%) and unmarried (87.5%). The average years of education were 5.5 years, and 55.0% of participants perceived their economic status as low. A total of 57.5% were living alone, and 45.0% perceived their health status as poor. The mean number of chronic diseases was 2.2. Additionally, 27.5% reported having oral health problems, 12.5% were current smokers, and 20.0% consumed alcohol at least once per month. Of the participants, 37.5% reported engaging in moderate-intensity physical activity. No statistically significant differences were found between the two groups across all general characteristics (Table 1).

**Table 1:** Participants’ general characteristics (*N* = 40)

Characteristics	*n* (%) or Mean (SD)			χ^2^ or t	*p*
	All (*N* = 40)	NurFP (*n* = 20)	Control (*n* = 20)		
**Sociodemographic**					
Age	77.7 (5.01)	77.3 (5.51)	78.2 (4.57)	-0.53	0.598
Gender					
Men	4 (10.0)	2 (10.0)	2 (10.0)	0.00	1.000
Women	36 (90.0)	18 (90.0)	18 (90.0)		
Marital status					
Married	5 (12.5)	3 (15.0)	2 (10.0)	0.23	0.633
Unmarried/divorced/widowed	35 (87.5)	17 (85.0)	18 (90.0)		
Education years	5.5 (3.69)	6.4 (4.21)	4.7 (2.94)	1.48	0.147
Perceived economic status					
High	18 (45.0)	8 (40.0)	10 (50.0)	0.40	0.525
Low	22 (55.0)	12 (60.0)	10 (50.0)		
Living alone					
Yes	23 (57.5)	11 (55.0)	12 (60.0)	0.10	0.749
No	17 (42.5)	9 (45.0)	8 (40.0)		
**Health-related**					
Self-rated health					
Good	22 (55.0)	9 (45.0)	13 (65.0)	4.69	0.096
Poor	18 (45.0)	11 (55.0)	7 (35.0)		
Chronic diseases, no	2.2 (0.86)	2.2 (0.81)	2.3 (0.92)	-0.52^a^	0.604
Oral health problems					
Yes	11 (27.5)	3 (15.0)	8 (40.0)	5.26	0.262
No	29 (72.5)	17 (85.0)	12 (60.0)		
Current smoking					
Yes	5 (12.5)	3 (15.0)	2 (10.0)	0.23	0.633
No	35 (87.5)	17 (85.0)	18 (90.0)		
Alcohol drinking					
≥ 1 time per month	8 (20.0)	2 (10.0)	6 (30.0)	2.50	0.114
< 1 time per month	32 (80.0)	18 (90.0)	14 (70.0)		
Exercise experience					
Yes	15 (37.5)	9 (45.0)	6 (30.0)	0.96	0.327
No	25 (62.5)	11 (55.0)	14 (70.0)		
Body mass index	25.4 (3.42)	26.4 (3.90)	24.4 (2.61)	1.84	0.073

SD = Standard deviation; ^a^Fisher’s exact test

### Effectiveness of the NurFP on frailty intrapersonal- and interpersonal-level factors

The NurFP and control groups were homogeneous in baseline primary and secondary outcome variables (Table 2). The primary outcome, frailty score, significantly decreased in the NurFP group (*p* = .003), whereas the control group showed a non-significant reduction. The between-group difference in mean differences scores was statistically significant (*p* = .025). In the physical frailty subdomain, scores significantly decreased in the NurFP group (*p* = .041), while no significant change was observed in the control group. The between-group difference was also statistically significant (*p* = .037). Regarding psychological frailty, the NurFP group showed a significant reduction (*p* = .002), while the control group exhibited a non-significant change. The between-group difference was not statistically significant. For social frailty, no significant reduction was observed in the NurFP group and control groups. However, the between-group difference was statistically significant (*p* = .020).

**Table 2:** Effects of a nurse-led frailty program on primary and secondary outcomes (*N* = 40)

	NurFP group (*n* = 20)				Control group (*n* = 20)				*p* ^b^
	Pretest	Posttest	Mean difference	*p* ^a^	Pretest	Posttest	Mean difference	*p* ^a^	
**Primary outcome**									
Frailty	8.4 (2.3)	7.1 (1.6)	-1.4 (1.6)	0.003	8.4 (2.5)	8.1 (2.3)	-0.4 (1.3)	0.287	0.025
Physical	3.9 (1.5)	3.4 (1.1)	-0.5 (1.1)	0.041	3.5 (2.1)	3.3 (1.9)	-0.2 (1.0)	0.480	0.037
Psychological	2.8 (1.2)	2.1 (0.9)	-0.7 (0.7)	0.002	3.1 (0.9)	2.8 (0.6)	-0.3 (0.7)	0.058	0.130
Social	1.8 (0.7)	1.6 (0.6)	-0.2 (0.5)	0.102	1.8 (1.2)	2.0 (1.0)	0.2 (0.4)	0.083	0.020
**Secondary outcomes**									
Intrapersonal-level									
Physical activity	79.3 (40.0)	124.8 (38.4)	45.5 (33.8)	< 0.001	73.8 (18.1)	71.0 (23.3)	-2.8 (13.5)	0.641	< 0.001
Dietary behavior	61.4 (10.5)	84.3 (36.5)	22.9 (36.6)	0.009	64.3 (12.7)	65.7 (13.4)	1.4 (17.3)	0.380	0.107
Self-management	40.6 (5.9)	47.9 (5.2)	7.3 (5.3)	< 0.001	42.7 (3.8)	41.7 (3.8)	1.0 (0.1)	0.100	<0.001
Interpersonal-level									
Social support	4.3 (1.5)	5.6 (1.2)	1.3 (0.6)	< 0.001	3.8 (1.9)	3.4 (1.5)	-0.4 (0.8)	0.046	< 0.001

^a^Wilcoxon signed-rank test for differences in changes from pretest and posttest within each group

^b^Mann-Whitney U test for differences in mean differences (values = posttest - pretest) between two groups

Homogeneity was confirmed by the Mann-Whitney U test for differences in pretest values between two groups

Among the secondary outcomes, the intrapersonal-level factor of adherence to physical activity significantly increased in the NurFP group (*p* < .001), while no significant change was observed in the control group. The between-group difference was statistically significant (*p* < .001). Adherence to healthy dietary behaviors also significantly improved in the NurFP group (*p* < .001), whereas the control group showed a slight but non-significant increase. The between-group difference was not statistically significant. Chronic disease self-management significantly improved in the NurFP group (*p* < .001), while no significant change was observed in the control group. The between-group difference was statistically significant (*p* < .001). Regarding the interpersonal-level factor, perceived social support significantly increased in the NurFP group (*p* < .001), while it significantly declined in the control group (*p* = .046). The between-group difference in change was also statistically significant (*p* < .001).

### Effectiveness of the NurFP on the frailty community-level factor

The FGIs conducted with study participants in the NurFP group, community health workers, and nurses revealed key insights into perceptions of the community-level frailty preventive and supportive environment of the program—namely, the creation of a supportive environment. Study participants expressed the multidisciplinary nature of the intervention, including the involvement of nurses, as fostering a sense of being respected and protected from health risk in the community. One participant stated, *“Having experts come directly to our local community service center to provide education made me feel respected and protected from health risk.”* Community health workers recognized the pivotal role of nurses in providing community health care, describing them as essential “health planners” in an aging society. One community health worker emphasized, *“This program made me realize how nurses can lead health planning*,*”* highlighting a supportive resource personnel environment for frailty prevention. They also perceived personal growth through their participation in the program, as reflected in the comment, *“It was a great learning experience for me.”* Nurses viewed the multidisciplinary educational support as reinforcing the feasibility of sustainable frailty prevention at the community level. As one nurse noted, *“Through this program*,* I felt that frailty prevention interventions could be sustainably implemented at the local community center level.”* This highlights the program’s potential for long-term integration within community health services targeting community-dwelling older populations.

## Discussion

We found that the NurFP group, compared to the control group, showed significant improvements not only in an overall score of frailty but also in its intrapersonal-, interpersonal-level frailty risk factors. Moreover, stakeholders involved in the NurFP program implementation—including intervention participants, community health workers, and nurses—recognized the program as a meaningful and empowering experience that fostered a supportive environment within the community and emphasized the importance of sustaining such initiatives as a model for community-based care that promotes respect, engagement, and personal growth. The NurFP program, a comprehensive intervention grounded in the ecological perspectives proposed by Salem et al. [16] and McLeroy et al. [20] may be effective among pre-frail older adults living in the community.

In this study, the NurFP program was found to be effective in reducing overall frailty scores, particularly in the physical and social domains. While previous studies have reported the effects of interventions on physical frailty [54, 55], few have examined frailty as a multidimensional construct and evaluated intervention outcomes across multiple domains. In our study, the significant improvements in physical and social frailty may be attributed to enhanced physical activity at the intrapersonal-level and group-based interventions, through providing participants opportunities for self-monitoring their physical activity and engaging them in structured, weekly 60-minute group walking sessions led by community health workers, fostering peer interaction and enhanced interrelationships. This group dynamics may also have played a role in alleviating physical and social frailty in the present study.

However, the NurFP program did not demonstrate effectiveness in the domain of psychological frailty. A recent systematic review [56] indicated that the effectiveness of frailty interventions—such as group-based exercise, case management, and music therapy—on psychological domains, including depression, remains inconclusive. While some studies have reported that group-based exercise interventions significantly reduce depressive symptoms among frail older adults [56], this finding was not replicated in the present study, despite incorporating similar group exercise sessions. This discrepancy may be attributed, in part, to the limited scope of the intervention in addressing cognitive function. The TFI instrument used in this study includes items assessing cognitive functioning, and 25.0% of participants presented with mild to moderate cognitive dysfunction at baseline. Given the nature of cognitive function— which often requires repeated, structured sessions and a prolonged intervention period to produce improvement [57]—and the lack of targeted cognitive training within the program, significant changes may not have been captured. Furthermore, the group-based delivery format may have lacked the depth needed to address individual symptoms of depression and psychological distress in a personalized manner.

In relation to intrapersonal-level factors, the present study confirmed the effectiveness of the NurFP program in promoting health behaviors. The intervention yielded significant improvements in adherence to physical activity among pre-frail older adults. This finding aligns with previous research by Yamada et al. [58], which demonstrated that initiating physical activity in later life can enhance functional independence and reduce mortality among older adults. These results underscore the importance of strategies that promote sustained physical activity in frail older populations, suggesting the utility of community resource-based approaches. Furthermore, consistent with Theou et al. [59], who identified engaging in physical activity two to three times per week as an optimal frequency for frailty prevention, the present study incorporated structured physical activity sessions with appropriate frequency and duration—specifically, a 40-minute weekly group exercise class led by a exercise specialist and a 60-minute weekly group walking session facilitated by community health workers—to support adherence to physical activity. These findings address that exercise interventions for frailty prevention should not be delivered as one-time programs but rather be designed to foster long-term behavioral engagement and maintenance.

We observed non-significant improvements in adherence to healthy dietary behaviors in the NurFP group compared to the control group. Although dietary behavior scores increased slightly in the intervention group, both groups demonstrated suboptimal adherence at baseline (approximately 60%) and failed to meet the recommended threshold (100%) after the intervention. Balanced protein intake at each meal has been shown to support muscle synthesis, physical function, and reduce the risk of falls and fractures, which are critical for frailty prevention [60–64]. However, challenges in translating nutrition education into sustained behavior change among older adults remain well documented [65]. A systematic review reported that group-based nutrition interventions for older adults often yield only modest effects unless accompanied by intensive behavioral strategies such as goal-setting, self-monitoring, and feedback [66]. Although a hands-on cooking session was included to enhance engagement and facilitate practical skill development, its limited frequency likely reduced its impact. Future interventions may benefit from incorporating more frequent and structured cooking sessions to strengthen dietary skills and promote sustainable behavior change.

In our findings, low self-management behaviors as an intrapersonal risk factor for frailty significantly improved in the NurFP group compared to the control group. There is empirical evidence that nurse-led individualized counseling and coaching can improve self-management behaviors among patients with chronic conditions such as diabetes [67], chronic low back pain [68], and coronary artery disease [69]. To our knowledge, this study is the first to evaluate the effect of nurse-led individualized counseling in the context of frailty as a complex condition. In particular, the NurFP program included weekly individualized counseling sessions, during which nurses conducted tailored assessments of each participant’s diverse symptoms related to chronic conditions and associated health behaviors. They also provided ongoing encouragement for self-monitoring and offered weekly evaluations and feedback on changes and progress in health behaviors. These components collectively contributed to enhancing participants’ self-directed engagement in managing chronic conditions. Meanwhile, given that chronic conditions cannot be resolved through short-term treatment and require ongoing management, enhancing individuals’ self-care capacity is critical for maintaining long-term health [70]. The NurFP program was designed based on previous literature [70] to address issues of chronic illness known to contribute to frailty, including falls, oral health problems, urinary incontinence, hypertension, diabetes, stroke, and arthritis. The program emphasized practical, actionable self-management strategies and fostered sustainable health management habits. Rather than relying solely on information delivery, this approach focused on empowering older adults with the skills and confidence to independently manage their health. As such, it represents an effective strategy that facilitates not only knowledge acquisition but also meaningful behavioral change.

With respect to interpersonal-level factors, the present study demonstrated the positive effect of the NurFP program on strengthening perceived social support among pre-frail older adults. This finding reinforces the significance of the intervention in line with a previous study by Ebrahimi et al. [71], which reported that limited social relationships and lack of social engagement in older adults are closely associated with feelings of loneliness, social isolation, and increased risk of frailty. Similarly, Fiori et al. [72] found that older adults with low levels of social participation exhibit poorer physical and mental health and have lower survival rates. The favorable outcomes observed in this study may be attributed to the consistent and proactive educational, emotional, and social support provided not only by community health nurses but also by community health workers. In addition, the group-based delivery of the program over 12 weeks likely facilitated the development of peer support among participants, further contributing to improved social connectedness. Given the relative scarcity of interventions targeting social support within frailty prevention programs for pre-frail older adults [73], these findings had uniqueness and also underscore the need for further research. Specifically, future community-based frailty interventions should incorporate structured strategies aimed at enhancing social support in order to better address the psychosocial vulnerabilities of aging populations.

In this study, perceptions of the supportive environment fostered by the NurFP program—as reported by intervention participants, community health nurses, and community health workers—suggest that the intervention played a role in fostering community capacity building. Participants expressed a sense of being respected and protected within their local context, while service providers such as nurses and community health workers recognized personal growth and strengthened professional capacity as a result of their involvement. These findings indicate that both the target population and health professionals benefited from the intervention. In practice, professionals delivering health services in community settings—particularly community health nurses—function not only as providers of care but also as human resources and organizational anchors within the community. Their enhanced competencies and professional satisfaction may be closely tied to service quality, which in turn supports behavioral change and health promotion among the population they serve [74]. This interpretation is consistent with findings from AK Draper, G Hewitt and S Rifkin [75], who reported that applying community capacity-building strategies in health promotion initiatives improved the health-related quality of life among home-dwelling older adults. Therefore, community capacity building can be regarded as an effective strategy for the systematic management of older adults’ health. In this study, the collaborative creation of a supportive environment—led by nurses in partnership with multidisciplinary experts and community health workers—played a pivotal role in the prevention of frailty.

The multidisciplinary approach employed in the NurFP intervention was likely a key factor in the observed improvements in frailty among participants. Given the multidimensional nature of frailty, an integrated and coordinated strategy is essential for effective prevention and management [76–78]. Pre-frailty, in particular, is considered a potentially reversible state, contingent upon timely and proactive engagement by individuals [76, 79]. Evidence suggests that providing appropriate interventions at the right time can serve as a cost-effective strategy for maintaining and promoting health among older adults [80]. A notable strength of the present study lies in its application of a multilevel and multidisciplinary framework to comprehensively address the health needs of pre-frail older adults in the community. In this regard, the study contributes meaningful insights into the development of evidence-based nursing practice, positioning community health nurses as central figures in the prevention and management of frailty among older adults.

Another consideration involves the potential influence of group effects on the study outcomes. The control group participants received a single health counseling session and otherwise continued their usual daily living in the community, whereas the intervention group was exposed to both individualized consultations and group sessions (i.e., group education and group walking sessions). The intervention program was implemented through weekly group sessions held at community health centers, where participants interacted with peers and community health nurses. Such group-based engagement may have enhanced participants’ motivation and adherence to frailty prevention behaviors through mutual encouragement and shared experiences. Nevertheless, the program also incorporated individualized consultations led by community health nurses at the beginning of each session, allowing participants to set personal goals and receive tailored feedback. Furthermore, participants continued to practice self-care independently in their home environment throughout the 12-week period. Therefore, the observed improvements likely reflect a combination of group dynamics, individualized nursing support, and self-directed behavior change rather than group influence alone. This mixed effect should be acknowledged when interpreting the intervention outcomes.

Several limitations of this study should be acknowledged. First, the study was conceptually guided by the ecological model, encompassing intrapersonal, interpersonal, and community levels of influence, as illustrated in the conceptual framework (Fig. 1). While the intervention incorporated community-level components—such as fostering a supportive environment for frailty prevention through community participation and collaboration with multiple health providers—the outcome evaluation primarily targeted individual and interpersonal outcomes. Furthermore, the limited operationalization of broader community-level and policy-related interventions, such as the enhancement of walkable neighborhood environments or the nationwide dissemination of frailty prevention services [51], may have constrained the full realization of the ecological approach. Future studies should strengthen multilevel integration by incorporating environmental and policy-level strategies, along with corresponding evaluation indicators, to improve both the scalability and sustainability of community-based frailty prevention programs. Second, the study was conducted among older adults residing in four administrative dongs in a district (among 25 districts in Seoul) in Seoul, South Korea. Therefore, caution is warranted when generalizing the findings to the broader older adult population. Third, the effectiveness of the program was evaluated by comparing pre-intervention data and post-intervention outcomes immediately after the 12-week program. As such, the long-term sustainability of the intervention effects remains unclear. Future studies should incorporate repeated measurements at extended follow-up points (e.g., 6 months, 1 year, or 2 years post-intervention) to assess whether the effects of the program are maintained over time. Fourth, the measure of adherence to healthy dietary behaviors developed by the researchers—a self-assessment checklist consisting of four items on protein intake embedded in participants’ meal diaries—requires validation in future studies. This is particularly important given the need for more comprehensive items that capture frailty-specific behavioral components of protein intake, beyond mere frequency. Moreover, the instrument we used to measure perceived social support—the Enhancing Recovery in Coronary Heart Disease Social Support Instrument—assessed support received from unspecified individuals. While the intervention did lead to improvements in perceived social support, it is likely that measuring support specifically from health care providers would have captured the intervention’s effects more sensitively. This should be taken into consideration in future research. Fifth, given that the intervention was implemented by practicing community health nurses within real-world community settings, the study was inherently designed as a pragmatic trial. Accordingly, the absence of blinded outcome assessors is a limitation that should be taken into account when interpreting the findings. Finally, another limitation concerns the absence of a double-blind study design. Because the study was conducted in real-world community settings, neither participants nor assessors were blinded to group allocation, which may have introduced potential observer bias or placebo effects. Future studies employing randomized controlled designs with partial or single blinding could further strengthen internal validity.

## Conclusions

This study demonstrated the effectiveness of the Nurse-led Frailty Prevention Program (NurFP)—a multilevel, integrated, and multidisciplinary intervention—in improving frailty and its associated ecological risk factors among community-dwelling prefrail older adults. Despite being at high risk for progressing to frailty, pre-frail individuals have received limited attention within the current healthcare system. These findings underscore the need for systematic community-based interventions targeting this vulnerable group. At the policy level, the NurFP serves as a model of a nurse-led, community-based program for frailty prevention, providing a practical example for standardization and nationwide dissemination through community health centers in Korea’s Visiting Health Management Program.

Furthermore, the findings highlight the pivotal role of nurses in leading community-based frailty prevention programs. As expert human resources, community health nurses are uniquely positioned to coordinate multidisciplinary teams, link community resources to meet the needs of older adults, conduct comprehensive assessments, and provide both group-based and individualized interventions. Their continuous engagement with community residents enables early identification of functional decline and timely, person-centered care. From a broader perspective, these findings indicate important policy implications for strengthening nurse-led frailty prevention services within community health systems. Collaboration with community health workers and mobilization of local resources can foster community participation, while institutional support from central and local governments is essential to expand and sustain the preventive and coordinating roles of community health nurses, thereby promoting healthy aging and reducing frailty at the population level.

## Supplementary Information

Below is the link to the electronic supplementary material.

Supplementary Material 1

## Data Availability

Data is available upon reasonable request. The data set used is available from the corresponding author upon reasonable request.

## Abbreviations

NurFP: Nurse-Led Frailty Prevention
MMSE-DS: Mini-Mental State Examination for Dementia Screening
TFI: Tilburg Frailty Indicator
FGI: focus group interview
