# Supplementary material for: Effectiveness of a nurse-led, community-based frailty prevention program for prefrail older adults: a pragmatic quasi-experimental trial
Source: BMC Nurs. 2025 Dec 12;25:62. doi: 10.1186/s12912-025-04131-4 (PMC12817711; doi:10.1186/s12912-025-04131-4)
Supplement: Supplementary file 1 — Supplementary Material 1 [file 12912_2025_4131_MOESM1_ESM.docx]

| Supplementary Table 1. Community characteristics of the four administrative dongs for group allocation | | | | | | |
| --- | --- | --- | --- | --- | --- | --- |
| **Community** | Population size | Prevalence of diagnosed disease | Self-rated health | Neighborhood environment | Health behavior | Housing type |
| Dong 1 | 8,878 | Low | Low | Low | Low | Detached house/Row house |
| Dong 2 | 9,495 | Low | Low | Low | Low | Detached house/Row house |
| Dong 3 | 11,197 | Moderate | Moderate | Moderate | Moderate | Apartment/Detached house |
| Dong 4 | 13,807 | Moderate | Moderate | Moderate | Moderate | Apartment/Detached house |
| Note. A 'Dong' is an administrative unit within a district in South Korea, similar to a neighborhood or precinct, used for local governance and public services | | | | | | |

| Supplementary Table 2. Professional training curriculum to enhance the competencies of community health nurses in frailty prevention within community settings | | | | |
| --- | --- | --- | --- | --- |
| Day | Ecological level | Topic | Educational content | Instructional method(s) |
| Day 1 | Introduction | Importance of comprehensive frailty prevention | - Challenges in frailty prevention among older adults in community settings - Review of frailty assessment tools and international best-practice manuals - Validity examination of early detection screening tools for frailty - - Structure and operation of comprehensive frailty prevention programs | Lecture, Discussion |
| Day 2 | Intrapersonal factors | Physical frailty prevention | - Appropriate exercise therapy for frailty prevention - Dietary guidelines for frailty prevention - Prevention and management of falls, oral health, medication use, and urinary incontinence - - Management of chronic conditions: hypertension, diabetes, stroke, and arthritis | Lecture, Demonstration, Discussion |
| Day 3 | Interpersonal factors | Psychological/Social frailty prevention | - Prevention of late-life depression and practice of depression screening tools - Methods and effects of operating health support groups - - How to guide and manage health diary use | Lecture, Discussion, hands-on Practice |
|  | Community-level factors | Community collaboration | - Analysis of challenges in nurse-led frailty prevention: self-assessment of competencies - Strategies for sustainable community-based frailty prevention: promoting community participation - Collaborative approaches to frailty management at the local level: multidisciplinary teamwork | Lecture, Discussion |
